# Neuron-periphery mitochondrial stress communication in aging and diseases

**DOI:** 10.1093/lifemedi/lnac051

**Published:** 2022-11-11

**Authors:** Jiasheng Li, Jimeng Cui, Ye Tian

**Affiliations:** State Key Laboratory of Molecular Developmental Biology, Institute of Genetics and Developmental Biology, Chinese Academy of Sciences, Beijing 100101, China; University of Chinese Academy of Sciences, Beijing 100093, China; State Key Laboratory of Molecular Developmental Biology, Institute of Genetics and Developmental Biology, Chinese Academy of Sciences, Beijing 100101, China; University of Chinese Academy of Sciences, Beijing 100093, China; State Key Laboratory of Molecular Developmental Biology, Institute of Genetics and Developmental Biology, Chinese Academy of Sciences, Beijing 100101, China; University of Chinese Academy of Sciences, Beijing 100093, China; Center for Excellence in Animal Evolution and Genetics, Chinese Academy of Sciences, Kunming 650223, China

**Keywords:** aging, neurodegeneration disease, mitochondria stress, inter-tissue communication, mitokine

## Abstract

The nervous system is the central hub of the body, detecting environmental and internal *stimuli* to regulate organismal metabolism via communications to the peripheral tissues. Mitochondria play an essential role in neuronal activity by supplying energy, maintaining cellular metabolism, and buffering calcium levels. A variety of mitochondrial conditions are associated with aging and age-related neurological disorders. Beyond regulating individual neuron cells, mitochondria also coordinate signaling in tissues and organs during stress conditions to mediate systemic metabolism and enable organisms to adapt to such stresses. In addition, peripheral organs and immune cells can also produce signaling molecules to modulate neuronal function. Recent studies have found that mitokines released upon mitochondrial stresses affect metabolism and the physiology of different tissues and organs at a distance. Here, we summarize recent advances in understanding neuron-periphery mitochondrial stress communication and how mitokine signals contribute to the systemic regulation of metabolism and aging with potential implications for therapeutic strategies.

## Introduction

In order to maintain organismal homeostasis and health, different tissues and organs inter-communicate their respective metabolic states via multiple signals. The nervous system is central to responding to environmental and internal *stimuli*, integrating and transmitting chemical or electrical signals to peripheral tissues for organismal-wide fitness [1–4]. Peripheral organs and the immune system can also produce bioactive factors that participate in inter-organ communication and affect the nervous system [5–9]. Hence, the bi-directional communication between neurons and peripheral tissues is essential for regulating organismal health and preventing the development of diseases.

Neurons are post-mitotic cells that, among other vital functions, are responsible for maintaining organismal homeostasis [4, 10]. Mitochondria are cellular organelles responsible for energy production and metabolism in cells, and thus provide the necessary energy supply for active neuronal synaptic transmission [11–13]. The intermediate metabolites derived from mitochondrial activity can affect neuronal gene expression due to epigenetic modifications [14]. In addition, mitochondrial stress signaling pathways mediate neuronal inflammation, calcium homeostasis, and inter-tissue stress communication [15, 16]. Mitochondrial deterioration within the nervous system is often associated with mutations in both nuclear and mitochondrial proteins, a decreased in mitochondrial oxidative phosphorylation (OXPHOS) activity, and an excessive production of mitochondrial reactive oxygen species (mtROS) [17, 18]. Dysregulated mitochondrial dynamic changes, including fusion and fission, also contribute to perturbed mitochondrial function and can lead to neurological disorders [19, 20]. Accordingly, it is essential for the nervous system to monitor and surveil mitochondrial function and activity through multiple quality control pathways [21, 22].

Mild mitochondrial proteostasis stress can activate mitochondrial unfolded protein responses (UPR^mt^), a transcriptional response that up-regulates the expression of mitochondrial chaperones, proteases, and detoxifying enzymes to help restore mitochondrial function [23–25]. In mammals, considerable evidence has highlighted the integrated stress response (ISR) as a central element of the UPR^mt^, where it differs from *Caenorhabditi elegans* [25–28]. If the damage is beyond repair, dysfunctional mitochondria are targeted for degradation via mitophagy, a form of autophagy that mediates the removal of defective/superfluous mitochondria from the cell [29, 30]. A delayed or deficient removal process can lead to the release of mitochondrial DNA (mtDNA) into the cytosol, where it may trigger immune response [31, 32]. In high-energy demanding neurons, the presence of damaged mitochondria may lead to neuronal cell death. Furthermore, age-associated mitochondrial dysfunction is highly correlated with many age-related neurological disorders, including Alzheimer’s disease (AD), Parkinson’s disease (PD), and amyotrophic lateral sclerosis (ALS) [13, 33].

The nervous system plays a central role in mediating the physiological function of peripheral tissues, whereby dysfunctional neuronal mitochondria can also compromise the homeostasis of peripheral tissues and overall organismal health, potentially leading to diseases development [20, 34]. A thorough understanding of the mitochondrial surveillance pathway and inter-organ mitochondrial stress communication under different physiological and pathological conditions is essential to provide therapeutic avenues for age-related neuronal diseases.

## The relationship between neuronal mitochondrial dysfunction, aging, and diseases

### Mitochondrial dysfunction in neurological disorders

Mitochondrial proteins are encoded by both the mtDNA and nuclear (nDNA) genomes, on which hundreds of mutations have been associated with a wide range of neurological disorders [18]. For example, high levels of the m.3243A > G heteroplasmy lead to mitochondrial dysfunction and a decrease in the number of synapses and dendritic complexity in humans. This results in mitochondrial encephalomyopathy, lactic acidosis, and stroke-like episodes (MELAS) syndrome [35]. Mutations in the optic atrophy 1 (OPAI) or Mitofusin 2 (MFN2) genes, which represent two mitochondrial dynamic regulators, lead to an impaired mitochondrial function and contribute to optic atrophy or charcot-marie-tooth disease type 2A (a familial neuropathy) [36, 37]. Moreover, mutations in the gene encoding pitrilysin metallopeptidase 1, a mitochondrial protease, lead to an age-dependent, progressive, neurological syndrome caused by the breakdown of mitochondrial precursors processing and degradation [38].

In mice, mitochondrial complex I (MCI) dysfunction in dopaminergic (DA) neurons results in progressive, human-like parkinsonism which decreases nigral dopamine release contributing to motor dysfunction [39]. Knockout of DRP1 in the forebrain of adult mice activates ISR and induces neuron-derived cytokine a fibroblast growth factor (FGF21), an early biomarker for latent neurodegenerative disorders [40]. Additionally, the excessive production of mtROS, mitophagy deficiency, or aberrant mitochondrial dynamics have also been associated with a variety of neuronal diseases (Table 1) [41–43].

**Table 1.** Mitochondria-associated neurologic disorders

Diseases	Mutation	Mt/nuclear encoded	Major functions of gene	Neuronal clinical feature	Age on set	Impaired mt dynamics/ morphology	Mt dysfunction	Experimental evidence	References
**MELAS**	High levels of m.3243A > G heteroplasmies in the MT-TL1 gene	mt	Code for tRNA^leu(UUR)^	Epilepsy, stroke-like episodes, intellectual and cortical sensory deficits, psychopathology (with muscle weakness, cardiomyopathy, and/or diabetes)	Adolescent/Adult/Childhood	Yes (reduced numbers)	Yes (Impaired mt OXPHOS)	High proportions of m.3243A > G shows induced neuronal cell death via mitochondrial respiratory dysfunction	[35, 44]
**DOA**	OPAI	Nuclear	Regulate mitochondrial fusion and energy output	Optic atrophy, progressive bilateral visual loss that begins early in life and extraocular neurological complications	Childhood	Yes (loss of mitochondrial fusion)	Yes (Impaired mt OXPHOS)	Cortical neurons OPA1 loss induces the pro-oxidative state, which may contribute to DOA pathogenesis	[37, 45]
**CMT2A**	MFN2	Nuclear	Dock and tether of neighboring mitochondria and outer membrane fusion	Sensory loss, degeneration of peripheral sensory and motor axons (with slowly progressive distal weakness and muscle atrophy)	Childhood/ Adult	Yes (abnormal mitochondrial transport)		MFN2 knockdown in spinal motor neurons exhibits axonal degeneration of spinal motor neurons, defection of mitochondrial morphology and function and other CMT2A disease-related phenotypes	[36, 46, 47]
**NARP**	m.8993TRG NARP mutation2 in the ATP synthase subunit 6 gene (MTATP6)	mt	Mitochondrial ATP synthesis coupled proton transport	impaired ability to coordinate voluntary movements (ataxia), migraines, seizures and learning disabilities (with developmental delays)	Childhood		Yes (abnormal mitochondrial energy generation)	T8993G mutation inhibits OXPHOS and results in enhanced free radical production in human cells, which might play an important role in the pathogenesis of NARP	[43, 48]
**MEMSA**	Polymerase gamma 1 (POLG)	Nuclear	mtDNA polymerase	Cerebellar ataxia, epilepsy, uncontrollable muscle jerks (myoclonus) and brain dysfunction (with muscle weakness)	Adolescent/Adult		Yes (fewer copies of mtDNA)		[49]
**SANDO**	POLG	nuclear	mtDNA polymerase	Sensory ataxia, seizures, and hearing loss (with myopathy)	Adult		Yes (mitochondrial dysfunction and mtDNA depletion in skeletal muscle and peripheral nerve) tissue	POLG W748S mutation in sf9 cells exhibits low DNA polymerase activity, low processivity and a severe DNA-binding defect	[41, 42]

### Age-related neuronal mitochondrial dysfunction

Neuronal mitochondrial function declines with age [20, 50–52], as exemplified by reduced mitochondria size and excessive mitochondrial damage in the brain of patients with aging-related disorders [53]. During aging, mitochondrial dysfunction is commonly associated with a decline in respiratory capacity (e.g., substrate-dependent oxygen consumption), an altered activity of mitochondrial enzymes (e.g., citrate synthase), an increased production of reactive oxygen species (ROS), and dysregulated mitochondrial proteostasis [54]. The altered mitochondrial function is also associated with changes in mitochondrial morphology. In *C. elegans*, mitochondria in neuron and body wall muscle cells exhibit progressive fragmentation during aging [50]. Notably, it has been reported that maintaining mitochondrial dynamics in the nervous system helps delay aging [55].

Since maintenance of an adequate number of mitochondria and homeostasis during the lifetime of neurons is critical for their development, connectivity, and plasticity, age-dependent neuronal mitochondrial function decline might, in turn, contribute to accelerating neurological disorders [50]. In mice, neuronal mitochondria synthesized the endogenous free radical scavenger melatonin, which is known to decrease with aging, resulting in the activation of neuronal inflammatory response [56]. Additionally, age-dependent mitochondrial loss or damage also leads to reduced ATP production, which accelerates both age-decline cerebral blood flow and mild cognitive impairment (MCI) [57]. Importantly, the overexpression of the gene cytochrome c oxidase subunit Va (COX5A) in neurons helps maintaining mitochondrial function and partly improves spatial recognition memory and hippocampal synaptic plasticity in aging mice [58].

## Neuron-to-periphery mitochondrial stress communication in diseases and aging

### Neuron-to-intestine communication

**Figure 1.**
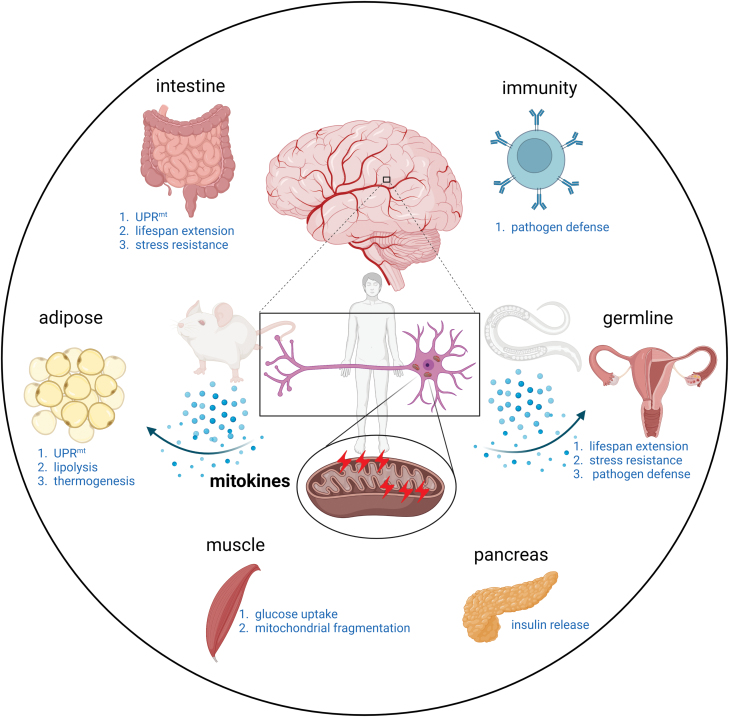
**Neuron-to-periphery mitochondrial stress communication.** In *C. elegans*, mice and humans, neurons under mitochondrial stress secrete mitokines to the peripheral tissues and organs, including the intestine, the WAT, the muscle, immune cells, the pancreas, and the germline. Peripheral tissues that perceived mitokines regulate systemic mitochondrial and metabolic responses and enable organisms to adapt to stress conditions.

The nervous system coordinates sensing and organismal-wide stress responses to ensure optimal fitness (Fig. 1) [16]. The gut of *C. elegans* serves not only as a digestive organ but also as a metabolic center, assembling liver, and adipose tissues in mammals, which makes it essential in the regulation of metabolism and aging. Durieux et al. found that neuron specific knockdown of a mitochondrial electron transfer chain complex IV component *cco-1(cox-5b)* resulted in robust induction of the UPR^mt^ in the intestine and extended lifespan in *C. elegans*. This study led to the formulation of the hypothesis that a secreted signaling molecule termed “mitokine,” which is released from the tissues/organs experiencing mitochondrial stresses, might be propagated and received by the distal tissues to induce mitochondrial stress response and adaptation [59]. Subsequently, further studies showed that various forms of mitochondrial dysfunction within the nervous system communicate stress status to the mitochondria in the intestine of *C. elegans*. This includes neuronal expression of the Huntington’s disease-causing polyglutamine expansion protein (Q40) [60], the expression of a mitochondrial outer membrane-targeted KillerRed [61], and the knockdown of the mt-AAA protease *spg-7* and mitochondrial fusion factor *fzo-1* [61, 62]. Recent studies reported that an enhanced GPCR signaling pathway in just two chemosensory ADL neurons is sufficient to induce intestinal UPR^mt^ and various physiological alterations in other peripheral tissues of *C. elegans* [63]. Interestingly, inter-tissue UPR^mt^ communication is essential for increased resistance to pathogenic bacterial infection, proteostasis stresses, and aging [59, 60].

The activation of the UPR^mt^ requires the transcriptional factor ATFS-1, DVE-1 and multiple epigenetic regulators, such as the histone methyltransferases LIN-65/MET-2, the histone demethylase JMJD-1.2/3.1, the histone deacetylase NuRD complex, and the histone acetyltransferase CBP-1 [14, 64–67]. In the absence of stress, the overexpression of JMJD-1.2/3.1 in the nervous system activates the induction of UPR^mt^ signals in the intestine and extends lifespan in *C. elegans* [65]. Additionally, mutations in the genes *baz-2* (an epigenetic regulator) and s*et-6* [a putative histone H3 lysine 9 (H3K9) methyltransferase], which are expressed in the nervous system, also elicit non-autonomous UPR^mt^ activation in the intestine. This, in turn, improves age-related complications and extends lifespan in *C. elegans* [68].

Despite recent advances, the study of the inter-tissue/organ UPR^mt^ signaling pathway in mammals is still in its infancy, and it is currently unknown whether mitochondrial perturbations within the nervous system also affect mitochondrial proteostasis of the intestine. Given that mitochondrial function is essential for neuronal activity, neuroendocrine signaling molecules could serve as mitokines to promote inter-organ mitochondrial stress communication and ensure organismal control of homeostasis.

### Neuron-to-white adipose tissue communication

Apart from the neuronal-intestine axis, homeostatic regulation of energy balance is also controlled by neuronal networks. Several studies showed that neuronal mitochondrial stress affects WAT metabolism. In mice, the deletion of OPA1, a master regulator of mitochondrial cristae remodeling, can cause severe mitochondrial dysfunction and alter mitochondrial Ca^2 + ^management in proopiomelanocortin (POMC) neurons [69]. In turn, mitochondrial dysfunction in POMC neurons leads to hyperphagia, the reduction of alpha-melanocyte stimulating hormone, attenuated lipolysis in white adipose tissue (WAT) and, finally, might cause obesity [70]. Another work reported that hypercaloric diets could induce obesity in partly due to microglia-hypersecretion of tumor-necrosis factor-α (TNFα) and mitochondrial dysfunction in the hypothalamic neurons [71]. The knockdown of TNFα downstream signals in the mediobasal hypothalamus reverses mitochondrial dysfunction and reduces body weight in high-fat diet (HFD)-induced obesity [71].

A different study evaluated knockout effects of the mitoribosome protein CR6-interacting factor 1(*Crif1*) in POMC neurons, and found it causes neuronal mitochondrial stress and obesity in mice [72, 73]. In contrast, mice with POMC-specific *Crif1* heterodeficiency show a high metabolic turnover with no alteration in body weight, lean, or fat mass. Further analysis suggested partial *Crif1* deficiency in POMC neurons activates thermogenesis and UPR^mt^ in adipose tissues by increasing the expression of β-endorphin (β-END) and mtDNA-encoded peptide MOTS-c (mitochondrial open reading frame of the 12S rRNA-c). MOTS-c upregulates Pomc transcription by STAT3 to coordinate mitoribosomal stress response in POMC neurons [72]. The metabolic discrepancies observed between knockout and knockdown *Crif1* in POMC neurons could be partly due to the extent/mode of mitochondrial stresses. Moreover, regular moderate-intensity exercise, like running, stimulates MOTS-c and β-END production in POMC neurons, indicating that hypothalamic neuronal mitohormesis may underlie exercise-induced high-turnover metabolism [72]. Finally, HFD-induced mice showed inhibition of mitochondrial dynamics, leptin sensitivity, and low-level glucose metabolism in POMC neurons. The knockout of apoptosis-inducing factor (AIF) in POMC neurons can increase fatty acid utilization and reverse mitochondrial dynamics, improving systemic glucose metabolism in obesity [74]. In light of these observations, the induction of mild mitochondrial stress in POMC neurons may serve as a strategy to treat HFD-induced obesity.

### Neuron-to-muscle communication

Since muscle cells are high-energy-demanding and highly-connected nerve cells, it is not surprising that neurons also communicate mitochondrial stress signals to the muscle. In *C. elegans*, neuronal *fzo-1* knockdown induces mitochondrial fragmentation in body wall muscles [62], which may affect the muscle cell physiology and organismal motility. In mice, HFD-fed POMC-*Cre*-dependent AIF deletion explored the relationship between neurons and glucose homeostasis in other tissues. Glucose uptake in skeletal muscle (SKM) was increased in HFD-fed AIF^ΔPOMC^ mice via improving mitochondrial morphology in POMC neurons [74]. These studies showed that the effects of neuronal-to-muscular mitochondrial communication are complex and highly influenced by dietary supplements and metabolic status.

### Neuron-to-immunity communication

The immune system is an important barrier to pathogens, and previous studies found that the innate immune response could be influenced by neuronal mitochondrial function. The overexpression of the mitochondrial chaperone HSP-60 in the nervous system increases the expression of innate immunity-related genes in the intestine, resulting in increased resistance against the pathogenic bacteria *Pseudomonas aeruginosa* (PA14) [75]. Similarly, neuronal *fzo-1* knockdown promotes resistance to pathogenic PA14 in *C. elegans* [62].

Mitochondrial dysfunction in DA neurons induced by the knockout of the mitochondrial fusion regulator MFN2 triggers an early onset immune response in adult midbrain DA neurons, which might exacerbate or drive neurodegenerative processes in adult-onset PD mice [76]. Another study revealed that the expression of mutant huntingtin disrupted mitochondrial function and led to the release of mitochondrial RNAs in neurons, activating innate immune signaling that correlates with vulnerability of striatal cells [77]. Furthermore, chemical inhibition of mitochondrial function induces Protein kinase R-mediated innate immune response in human neurons [77]. Accordingly, the mitochondrial signaling crosstalk between neurons and the immune system is a growing area of neurodegenerative diseases research.

### Neuron-to-pancreas communication

Since mitochondria serve as major energy production factories within the cell and represent a communication hub for adaptive stress responses, mitochondrial dysfunction might lead to the downstream disruption of insulin signaling and an accumulation of toxic lipids, which can be potentially connected to obesity and a predisposition to type 2 diabetes mellitus [78]. It has been demonstrated that the mitochondrial fusion proteins MFN1 in POMC neurons are required for dynamic remodeling of mitochondrial networks in response to metabolic challenges, central glucose sensing, and insulin release from the pancreas. Specifically, *Mfn1* deletion causes reduced mitochondrial cytosol coverage area in POMC neurons, leading to defective pancreatic insulin release by enhanced central ROS production [79]. However, this would suggest that mitochondrial dynamics in POMC neurons serve as an intrinsic nutrient-sensing mechanism.

### Neuron-to-germline communication

Interestingly, mitochondrial stress response is transmitted not only between neurons and somatic tissues, but also occurs between the germline and thus across generations, with potential physiological implication for the offspring [80]. A study from Zhang et al. found that the systemic activation of UPR^mt^ caused by neuronal mitochondrial perturbations can be transmitted to descendants over multiple generations in *C. elegans*. The memory of neuronal mitochondrial stress is passed down to the offspring via maternal inheritance of elevated levels of mtDNA, resulting in an imbalance of the mtDNA/nDNA ratio, and leading to mitochondrial proteostasis stress in each generation [81]. The offspring affected by transgenerational mitochondrial stress responses exhibit increased resistance to stress and longer lifespans, despite showing a slightly delayed development and reduced brood size. While this mechanism remains elusive in mammals, elevated mtDNA levels directly contribute to health in other organisms.

## Periphery-to-neuron mitochondrial stress communication in diseases and aging

### Intestine-to-neuron communication

**Figure 2.**
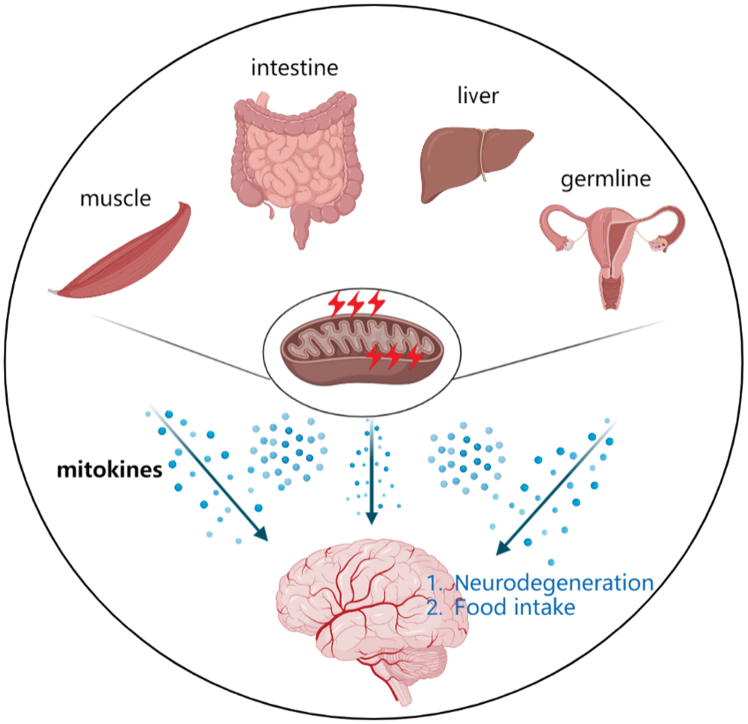
**Periphery-to-neuron mitochondria stress communication.** Peripheral organs under mitochondrial stress secrete mitokines and affect neuronal function. The inflammation and metabolism of the intestine, liver, and muscle affected by mitochondrial function can also communicate the stress signal with neurons, suppressing sugar intake and potentially protecting agaist neurodegeneration.

Although neuronal disorders have traditionally been considered a consequence of processes autonomous to vulnerable neurons, it has become increasingly recognized that non-autonomous cellular mechanisms influence neuronal degeneration (Fig. 2). The intestine is a tissue that directly contacts the external environment, whereby it is often affected by environmental conditions, and communicates with neurons [82]. In *C. elegans*, rotenone is a widely used mitotoxin that causes MCI dysfunction [83, 84]. A previous study found that the p38MAPK/CREB/ATF-7-dependent innate immune response pathway in intestinal cells was activated by rotenone exposure, protecting rotenone-induced DA neurodegeneration through mitophagy [5]. However, the mechanisms through which p38MAPK and/or ATF-7 regulate mitophagy in *C. elegans* and the transmission of signals between tissues remain elusive.

It has been previously demonstrated that impaired systemic mitophagy induces an increase in serum inflammatory cytokines, contributing to DA neurodegeneration [85]. An intestine-to-neuron mitochondrial communication was also observed in *Drosophila*. Mutations in the gene encoding the mitochondrial PTEN-induced kinase1 (PINK1) lead to an accumulation of defective mitochondria and cause a form of familial PD, an age-related neurodegenerative diseases [86]. Surprisingly, neuronal defects in *pink1*-mutant flies can be rescued by genetic and pharmacological inhibition of the immune response in the intestine [6]. It is also worth noting that neuronal disorders associated with deficient mitophagy machinery in flies are modulated by a non-autonomous cellular signaling pathway induced by mitochondrial toxicity in the intestine [6]. Furthermore, motor impairment can be aggravated by intestinal inflammation caused by *Citrobacter rodentium* infection, as shown in a PD-based *Pink1* knockout mice model. Together, these studies reinforce the link between gut inflammation and neurodegeneration in worms, files, and mice. This provides a solid foundation to explore the underlying mechanisms of intestinal inflammation signals to the nervous system, and develop therapeutic strategies for neurodegenerative diseases via interventions in the intestine.

### Liver-to-neuron communication

A growing amount of evidence showed that the nervous system and cognitive function are adversely affected by obesity. For example, obesity-induced diabetic mice exhibit a disruption in neuronal mitochondrial homeostasis [87], suggesting that changes in liver mitochondrial function directly affect neuronal function. Additionally, the liver-specific deletion of the mitochondrial fission regulator *Drp1* induces the expression of FGF21, a hormone that is secreted by the liver, which in turn increases energy expenditure and protects animals against HFD-induced obesity [88]. Moreover, liver derived FGF21 enhances glucose responsiveness of the ventromedial hypothalamus to suppress sugar intake [89]. The mtDNA polymerase POLG mutator mice is also able to induce FGF21 expression [90], and young POLG mice placed on a HFD are completely resistant to diet-induced obesity [90]. Remarkably, the overexpression of FGF21 in hepatocytes increases mice lifespan [91], but it is still unclear whether liver-nerve mitochondrial communication can be used to develop therapeutic targets for metabolic diseases.

### Muscle-to-neuron communication

In-depth understanding of the muscle-brain axis showed that regular exercise not only enhances mitochondrial function in SKM but also benefits the brain and is a major protective factor against neurodegenerative diseases [92]. The gene *Ucp1* compromises mitochondrial OXPHOS capacity via respiratory uncoupling. Mice with muscle-specific overexpression of *Ucp1* show SKM-specific induction and diurnal variation in the release of the growth differentiation factor (GDF15) [93]. Muscle-derived GDF15 promotes diurnal anorexia during mitochondrial dysfunction. GDF15 regulates food intake and body weight in the area postrema and the nucleus of solitary tract neurons [94, 95]. In humans, aerobic fitness (endurance performance) is negatively correlated with the loss of nervous tissue and cognitive deterioration with age, and restoration of mitochondrial function through physical exercise delays the onset and slows the progression of AD [96]. Accordingly, interventions on muscle metabolism might improve neuronal function that can be employed to treat neurodegenerative diseases.

### Germline-to-neuron communication

In *C. elegans*, germline-specific proteostasis perturbation not only results in mitochondrial network alteration in the germline, but also triggers the aggregation of various disease-causing proteins including polyQ67, ALS-related mutant variants of FUS, and TDP-43 in neurons through the action of the mitokine Wnt/EGL-20 signal [97]. Whether the germline-to-neuron stress communication is present in other organisms remains to be resolved.

## The mitokine signals

**Figure 3.**
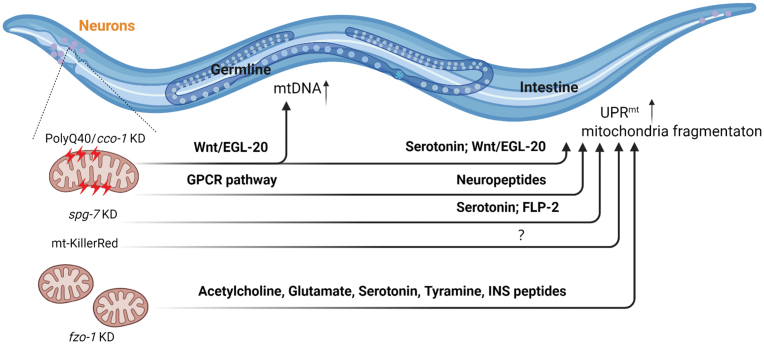
**Mitokines signaling communication of neuronal mitochondrial stress in *C. elegans*.** The different types of neuronal mitochondrial stress communication and associated mitokines ensure communication between the nervous system and peripheral organs in *C. elegans*. Serotonin, Wnt/EGL-20, or FLP-2 are required for UPR^mt^ activation in the intestine in response to neuronal *cco-1(cox-5b)/spg-7* knockdown or neuronal polyQ40 expression. Activation of UPR^mt^ in the intestine upon loss of *fzo-1* in the neurons requires multiple neurotransmitters and neuropeptides, including acetylcholine, tyramine, glutamate, serotonin, and two insulin-like peptides, INS-27 and INS-35. Activation of GPCR signaling in just two sensory neurons is sufficient to activate intestinal UPR^mt^ via releasing neuropeptides.

The inter-organ mitochondrial stress communication requires signal molecules, in particular mitokine generated from cells undergoing mitochondrial stress, which are then released and perceived in receiving cells for organismal-wide stress adaptation [16]. Even though the nature of mitokine molecules remains largely elusive, several secreted factors are known to facilitate mitokine signaling transduction across different tissues (Fig. 3) [98, 99].

In *C. elegans*, neurotransmitter serotonin is required for non-autonomous cellular UPR^mt^ activation in the intestine in response to neuronal *cco-1(cox-5b)/spg-7* knockdown or neuronal polyQ40 expression [60]. However, the exogenous supplementation of serotonin is not sufficient to activate the UPR^mt^ in the intestine of *C. elegans*, suggesting that serotonin does not act alone [60]. In addition, the neuropeptide FLP-2 is also required for non-autonomous cellular UPR^mt^ in a neuronal *spg-7* deletion model [61]. However, the overexpression of FLP-2 in neurons is sufficient to induce UPR^mt^ in the intestine without affecting the lifespan of animals, suggesting other pro-longevity mitokine molecules remain unidentified [61]. Recently, it has been demonstrated that multiple neurotransmitters and neuropeptides are required for neuronal *fzo-1* loss-induced non-autonomous cellular UPR^mt^ in the intestine, including acetylcholine, tyramine, glutamate, serotonin, and two insulin-like peptides, INS-27 and INS-35 [62].

A genetic screen identified that loss-of-function mutations of retromer complex components responsible for recycling the Wnt secretion-factor/MIG-14 suppress the neuron-to-intestine mitochondrial proteotoxic stress communication [100]. Furthermore, the overexpression of the Wnt ligand EGL-20 within the nervous system activates the mitochondrial stress response pathway in the intestine, resulting in organismal-wide metabolic adaptations and lifespan extension [100]. This also allowed to identify a disulfide isomerase, PDI-6, which interacts with Wnt ligand/EGL-20 in the endoplasmic reticulum through disulfide-dependent association. PDI-6 stabilizes Wnt/EGL-20 protein and facilitate Wnt secretion for the propagation of cell non-autonomous mitochondrial stress signaling. The neuronal overexpression of PDI-6 can also coordinate organismal mitochondrial proteostasis stress and increase lifespan in a Wnt-dependent manner [101].

In mice, a peptide hormone synthesized by multiple organs (FGF21) is induced during muscle stress and mitochondrial myopathies to help increase energy expenditure and regulate the metabolism of organs/tissues non-autonomously [102]. Similarly, GDF15 is identified as a mammalian mitokine due to its role in mitochondrial ISR, which regulates metabolism by suppressing food intake and controlling energy intake, and prevents diet-induced hepatic steatosis [103]. In addition, several mitochondrial-derived peptides play a role in inter-organ mitochondrial stress communication, including MOTS-c, Humanin, Small humanin-like peptides 2/3, Adrenomedullin 2, and Angiopoietin-like 6 [104–108]. Importantly, the mechanisms by which these mitokine molecules are produced in tissues/organs experiencing mitochondrial stresses, and are subsequently released, propagated, and finally perceived by the targeted tissues/organs deserves further exploration, specifically regarding systemic mitochondrial and metabolic regulation in various diseases and the aging process.

## Conclusions

Mitochondria engulfing promoted the evolution from single cells to complex multicellular systems that require inter- and intracellular coordination and metabolism to grow and survive under various environmental conditions. Apart from the established roles in bioenergetics and biosynthesis, mitochondrial signaling actively regulates organismal health and aging [109]. The nervous system is essential for sensing, integrating, and transmitting information to peripheral tissues (e.g., Liver, muscle, intestine) for optimal fitness, but the metabolic changes associated with these processes remain poorly understood. Despite the importance of establishing non-autonomous cellular mitochondrial stress regulation in model organisms, it is also crucial to examine the conservation of these inter-tissue mitochondrial signaling networks in complex mammals. In particular, it is important to understand how key signaling tissues integrate stress stimuli and orchestrate organismal-wide metabolic adaptations via mitokine signals, and how this could be employed to develop therapeutic strategies to treat metabolic diseases.

## Perspectives

Over the course of evolution, from unicellular organisms to multicellular organisms, had developed mechanisms for sensing and responding to internal and extracellular changes in the environment. Intercellular communication is further complicated in multicellular organisms, as different tissues would need to cooperate extensively as a whole system and balance distinctive metabolic states of different cell types in the normal state and under stress conditions. As the system becomes more complex, intercellular communication will evolve to achieve an increased level of complexity. Thus, it is interesting to explore whether the mitokine signals identified in *C. elegans* have similar roles in mammals. Are different mitokines act alone or synergistically to meet diverse physiological needs? The more important question is to understand how the mitokine signal is precisely regulated during either temporary or long-term mitochondrial stress conditions. Whether the effects of systemic mitochondrial stress communication are beneficial or detrimental for an organism under various stress conditions or during the aging process. The establishment of more complex systems in different model organisms, stereoscopic cell co-culture assays, and human organoids are in need to expedite the discoveries in the field.

## References

[1] Van der Kolk BA. Clinical implications of neuroscience research in PTSD. Ann N Y Acad Sci 2006;1071:277–93. 16891578 10.1196/annals.1364.022

[2] Li S, Sheng ZH. Energy matters: presynaptic metabolism and the maintenance of synaptic transmission. Nat Rev Neurosci 2022;23:4–22. 34782781 10.1038/s41583-021-00535-8

[3] Carter R. The Human Brain Book: An Illustrated Guide to its Structure, Function, and Disorders. London: Penguin, 2019.

[4] Sudhof TC. Towards an understanding of synapse formation. Neuron 2018;100:276–93. 30359597 10.1016/j.neuron.2018.09.040 PMC6226307

[5] Chikka MR, Anbalagan C, Dvorak K, et al. The mitochondria-regulated immune pathway activated in the C. elegans intestine is neuroprotective. Cell Rep 2016;16:2399–414. 27545884 10.1016/j.celrep.2016.07.077 PMC7780887

[6] Fedele G, Loh SHY, Celardo I, et al. Suppression of intestinal dysfunction in a Drosophila model of Parkinson’s disease is neuroprotective. Nat Aging 2022;2:317–31. 37117744 10.1038/s43587-022-00194-z

[7] Matheoud D, Cannon T, Voisin A, et al. Intestinal infection triggers Parkinson’s disease-like symptoms in Pink1(-/-) mice. Nature 2019;571:565–9. 31316206 10.1038/s41586-019-1405-y

[8] Savini M, Folick A, Lee Y-T, et al. Lysosome lipid signalling from the periphery to neurons regulates longevity. Nat Cell Biol 2022;24:906–16. 35681008 10.1038/s41556-022-00926-8 PMC9203275

[9] Dantzer R. Neuroimmune interactions: from the brain to the immune system and vice versa. Physiol Rev 2018;98:477–504. 29351513 10.1152/physrev.00039.2016 PMC5866360

[10] Tooze SA, Schiavo K. Liaisons dangereuses: autophagy, neuronal survival and neurodegeneration. Curr Opin Neurobiol 2008;18:504–15. 18840524 10.1016/j.conb.2008.09.015

[11] van der Bliek AM, Sedensky MM, Morgan PG. Cell biology of the mitochondrion. Genetics 2017;207:843–71. 29097398 10.1534/genetics.117.300262 PMC5676242

[12] Yellen G. Fueling thought: management of glycolysis and oxidative phosphorylation in neuronal metabolism. J Cell Biol 2018;217:2235–46. 29752396 10.1083/jcb.201803152 PMC6028533

[13] Cheng XT, Huang N, Sheng ZH. Programming axonal mitochondrial maintenance and bioenergetics in neurodegeneration and regeneration. Neuron 2022;110:1899–923. 35429433 10.1016/j.neuron.2022.03.015 PMC9233091

[14] Zhu D, Wu X, Zhou J, et al. NuRD mediates mitochondrial stress-induced longevity via chromatin remodeling in response to acetyl-CoA level. Sci Adv 2020;6:eabb2529. 32789178 10.1126/sciadv.abb2529 PMC7400466

[15] Anderson AJ, Jackson TD, Stroud DA, et al. Mitochondria-hubs for regulating cellular biochemistry: emerging concepts and networks. Open Biol 2019;9:190126. 31387448 10.1098/rsob.190126 PMC6731593

[16] Bar-Ziv R, Bolas T, Dillin A. Systemic effects of mitochondrial stress. EMBO Rep 2020;21:e50094. 32449292 10.15252/embr.202050094 PMC7271648

[17] Zhu D, Li X, Tian Y. Mitochondrial-to-nuclear communication in aging: an epigenetic perspective. Trends Biochem Sci 2022;47:645–59. 35397926 10.1016/j.tibs.2022.03.008

[18] Chow J, Rahman J, Achermann JC, et al. Mitochondrial disease and endocrine dysfunction. Nat Rev Endocrinol 2017;13:92–104. 27716753 10.1038/nrendo.2016.151

[19] Mishra P, Chan DC. Mitochondrial dynamics and inheritance during cell division, development and disease. Nat Rev Mol Cell Biol 2014;15:634–46. 25237825 10.1038/nrm3877 PMC4250044

[20] Su B, Wang X, Zheng L, et al. Abnormal mitochondrial dynamics and neurodegenerative diseases. Biochim Biophys Acta 2010;1802:135–42. 19799998 10.1016/j.bbadis.2009.09.013 PMC2790543

[21] Gao F, Zhang J. Mitochondrial quality control and neurodegenerative diseases. Neuronal Signal 2018;2:NS20180062. 32714594 10.1042/NS20180062 PMC7373240

[22] Tan JX, Finkel T. Mitochondria as intracellular signaling platforms in health and disease. J Cell Biol 2020;219:e202002179. 32320464 10.1083/jcb.202002179 PMC7199861

[23] Haynes CM, Ron D. The mitochondrial UPR - protecting organelle protein homeostasis. J Cell Sci 2010;123:3849–55. 21048161 10.1242/jcs.075119

[24] Shpilka T, Haynes CM. The mitochondrial UPR: mechanisms, physiological functions and implications in ageing. Nat Rev Mol Cell Biol 2018;19:109–20. 29165426 10.1038/nrm.2017.110

[25] Anderson NS, Haynes CM. Folding the mitochondrial UPR into the integrated stress response. Trends Cell Biol 2020;30:428–39. 32413314 10.1016/j.tcb.2020.03.001 PMC7230072

[26] Fiorese CJ, Schulz AM, Lin YF, et al. The transcription factor ATF5 mediates a mammalian mitochondrial UPR. Curr Biol 2016;26:2037–43. 27426517 10.1016/j.cub.2016.06.002 PMC4980197

[27] Quiros PM, Prado MA, Zamboni N, et al. Multi-omics analysis identifies ATF4 as a key regulator of the mitochondrial stress response in mammals. J Cell Biol 2017;216:2027–45. 28566324 10.1083/jcb.201702058 PMC5496626

[28] Forsstrom S, Jackson CB, Carroll CJ, et al. Fibroblast growth factor 21 drives dynamics of local and systemic stress responses in mitochondrial myopathy with mtDNA deletions. Cell Metab 2019;30:1040–54 e1047. 31523008 10.1016/j.cmet.2019.08.019

[29] East DA, Campanella M. Mitophagy and the therapeutic clearance of damaged mitochondria for neuroprotection. Int J Biochem Cell Biol 2016;79:382–7. 27586258 10.1016/j.biocel.2016.08.019

[30] Youle RJ, Narendra DP. Mechanisms of mitophagy. Nat Rev Mol Cell Biol 2011;12:9–14. 21179058 10.1038/nrm3028 PMC4780047

[31] Riley JS, Tait SW. Mitochondrial DNA in inflammation and immunity. EMBO Rep 2020;21:e49799. 32202065 10.15252/embr.201949799 PMC7132203

[32] West AP, Shadel GS. Mitochondrial DNA in innate immune responses and inflammatory pathology. Nat Rev Immunol 2017;17:363–75. 28393922 10.1038/nri.2017.21 PMC7289178

[33] Han Q, Xu XM. Mitochondrial integrity in neuronal injury and repair. Neural Regen Res 2021;16:674–5. 33063720 10.4103/1673-5374.295317 PMC8067945

[34] Mottis A, Herzig S, Auwerx J. Mitocellular communication: shaping health and disease. Science 2019;366:827–32. 31727828 10.1126/science.aax3768

[35] Klein Gunnewiek TM, Van Hugte EJH, Frega M, et al. m.3243A > G-induced mitochondrial dysfunction impairs human neuronal development and reduces neuronal network activity and synchronicity. Cell Rep 2020;31:107538. 32320658 10.1016/j.celrep.2020.107538

[36] Kijima K, Numakura C, Izumino H, et al. Mitochondrial GTPase mitofusin 2 mutation in Charcot-Marie-Tooth neuropathy type 2A. Hum Genet 2005;116:23–7. 15549395 10.1007/s00439-004-1199-2

[37] Zanna C, Ghelli A, Porcelli AM, et al. OPA1 mutations associated with dominant optic atrophy impair oxidative phosphorylation and mitochondrial fusion. Brain 2008;131:352–67. 18222991 10.1093/brain/awm335

[38] Perez MJ, Ivanyuk D, Panagiotakopoulou V, et al. Loss of function of the mitochondrial peptidase PITRM1 induces proteotoxic stress and Alzheimer’s disease-like pathology in human cerebral organoids. Mol Psychiatry 2021;26:5733–50. 32632204 10.1038/s41380-020-0807-4 PMC8758476

[39] Gonzalez-Rodriguez P, Zampese E, Stout KA, et al. Disruption of mitochondrial complex I induces progressive parkinsonism. Nature 2021;599:650–6. 34732887 10.1038/s41586-021-04059-0 PMC9189968

[40] Restelli LM, Oettinghaus B, Halliday M, et al. Neuronal mitochondrial dysfunction activates the integrated stress response to induce fibroblast growth factor 21. Cell Rep 2018;24:1407–14. 30089252 10.1016/j.celrep.2018.07.023 PMC6092266

[41] Chan SS, Longley MJ, Copeland WC. Modulation of the W748S mutation in DNA polymerase gamma by the E1143G polymorphismin mitochondrial disorders. Hum Mol Genet 2006;15:3473–83. 17088268 10.1093/hmg/ddl424 PMC1780027

[42] Fadic R, Russell JA, Vedanarayanan VV, et al. Sensory ataxic neuropathy as the presenting feature of a novel mitochondrial disease. Neurology 1997;49:239–45. 9222196 10.1212/wnl.49.1.239

[43] Mattiazzi M, Vijayvergiya C, Gajewski CD, et al. The mtDNA T8993G (NARP) mutation results in an impairment of oxidative phosphorylation that can be improved by antioxidants. Hum Mol Genet 2004;13:869–79. 14998933 10.1093/hmg/ddh103

[44] Yokota M, Hatakeyama H, Ono Y, et al. Mitochondrial respiratory dysfunction disturbs neuronal and cardiac lineage commitment of human iPSCs. Cell Death Dis 2017;8:e2551. 28079893 10.1038/cddis.2016.484 PMC5386384

[45] Millet AM, Bertholet AM, Daloyau M, et al. Loss of functional OPA1 unbalances redox state: implications in dominant optic atrophy pathogenesis. Ann Clin Transl Neurol 2016;3:408–21. 27547769 10.1002/acn3.305 PMC4891995

[46] Baloh RH, Schmidt RE, Pestronk A et al. Altered axonal mitochondrial transport in the pathogenesis of Charcot-Marie-Tooth disease from mitofusin 2 mutations. J Neurosci 2007;27:422–30. 17215403 10.1523/JNEUROSCI.4798-06.2007 PMC6672077

[47] Mou Y, Dein J, Chen Z, et al. MFN2 deficiency impairs mitochondrial transport and downregulates motor protein expression in human spinal motor neurons. Front Mol Neurosci 2021;14:727552. 34602978 10.3389/fnmol.2021.727552 PMC8482798

[48] https://www.orpha.net/consor/cgi-bin/Disease_Search.php?lng=EN&data_id=182&Disease_Disease_Search_diseaseGroup=NARP&Disease_Disease_Search_diseaseType=Pat&Disease(s)/group%20of%20diseases=NARP-syndrome&title=NARP%20syndrome&search=Disease_Search_Simple.

[49] https://medlineplus.gov/genetics/condition/myoclonic-epilepsy-myopathy-sensory-ataxia/.

[50] Jiang HC, Hsu JM, Yen CP, et al. Neural activity and CaMKII protect mitochondria from fragmentation in aging Caenorhabditis elegans neurons. Proc Natl Acad Sci USA 2015;112:8768–73. 26124107 10.1073/pnas.1501831112 PMC4507213

[51] Short KR, Bigelow ML, Kahl J, et al. Decline in skeletal muscle mitochondrial function with aging in humans. Proc Natl Acad Sci USA 2005;102:5618–23. 15800038 10.1073/pnas.0501559102 PMC556267

[52] Cai Y, Song W, Li J, et al. The landscape of aging. Sci China Life Sci 2022; doi:10.1007/s11427-022-2161-3. PMC9446657 36066811

[53] Fang EF, Hou Y, Palikaras K, et al. Mitophagy inhibits amyloid-beta and tau pathology and reverses cognitive deficits in models of Alzheimer’s disease. Nat Neurosci 2019;22:401–12. 30742114 10.1038/s41593-018-0332-9 PMC6693625

[54] Sun N, Youle RJ, Finkel T. The mitochondrial basis of aging. Mol Cell 2016;61:654–66. 26942670 10.1016/j.molcel.2016.01.028 PMC4779179

[55] Weir HJ, Yao P, Huynh FK, et al. Dietary restriction and AMPK increase lifespan via mitochondrial network and peroxisome remodeling. Cell Metab 2017;26:884–96 e885. 29107506 10.1016/j.cmet.2017.09.024 PMC5718936

[56] Jauhari A, Baranov SV, Suofu Y, et al. Melatonin inhibits cytosolic mitochondrial DNA-induced neuroinflammatory signaling in accelerated aging and neurodegeneration. J Clin Invest 2020;130:3124–36. 32182222 10.1172/JCI135026 PMC7260019

[57] de la Torre J C., Olmo A. D, Valles S. Can mild cognitive impairment be stabilized by showering brain mitochondria with laser photons? Neuropharmacology 2020;171:107841. 31704275 10.1016/j.neuropharm.2019.107841

[58] Xiyang YB, Liu R, Wang XY, et al. COX5A plays a vital role in memory impairment associated with brain aging via the BDNF/ERK1/2 signaling pathway. Front Aging Neurosci 2020;12:215. 32754029 10.3389/fnagi.2020.00215 PMC7365906

[59] Durieux J, Wolff S, Dillin A. The cell-non-autonomous nature of electron transport chain-mediated longevity. Cell 2011;144:79–91. 21215371 10.1016/j.cell.2010.12.016 PMC3062502

[60] Berendzen KM, Durieux J, Shao LW, et al. Neuroendocrine coordination of mitochondrial stress signaling and proteostasis. Cell 2016;166:1553–63 e1510. 27610575 10.1016/j.cell.2016.08.042 PMC5922979

[61] Shao LW, Niu R, Liu Y. Neuropeptide signals cell non-autonomous mitochondrial unfolded protein response. Cell Res 2016;26:1182–96. 27767096 10.1038/cr.2016.118 PMC5099867

[62] Chen LT, Lin CT, Lin LY, et al. Neuronal mitochondrial dynamics coordinate systemic mitochondrial morphology and stress response to confer pathogen resistance in C. elegans. Dev Cell 2021;56:1770–85 e1712. 33984269 10.1016/j.devcel.2021.04.021

[63] Liu Y, Zhou J, Zhang N, et al. Two sensory neurons coordinate the systemic mitochondrial stress response 2 via GPCR signaling in C. elegans. Dev Cell 2022;57:2469–82. 36309009 10.1016/j.devcel.2022.10.001

[64] Tian Y, Garcia G, Bian Q, et al. Mitochondrial stress induces chromatin reorganization to promote longevity and UPR(mt). Cell 2016;165:1197–208. 27133166 10.1016/j.cell.2016.04.011 PMC4889216

[65] Merkwirth C, Jovaisaite V, Durieux J, et al. Two conserved histone demethylases regulate mitochondrial stress-induced longevity. Cell 2016;165:1209–23. 27133168 10.1016/j.cell.2016.04.012 PMC4889222

[66] Li TY, Sleiman MB, Li H, et al. The transcriptional coactivator CBP/p300 is an evolutionarily conserved node that promotes longevity in response to mitochondrial stress. Nat Aging 2021;1:165–78. 33718883 10.1038/s43587-020-00025-z PMC7116894

[67] Shao LW, Peng Q, Dong M, et al. Histone deacetylase HDA-1 modulates mitochondrial stress response and longevity. Nat Commun 2020;11:4639. 32934238 10.1038/s41467-020-18501-w PMC7493924

[68] Yuan J, Chang SY, Yin SG, et al. Two conserved epigenetic regulators prevent healthy ageing. Nature 2020;579:118–22. 32103178 10.1038/s41586-020-2037-y

[69] Cipolat S, Rudka T, Hartmann D, et al. Mitochondrial rhomboid PARL regulates cytochrome c release during apoptosis via OPA1-dependent cristae remodeling. Cell 2006;126:163–75. 16839884 10.1016/j.cell.2006.06.021

[70] Gomez-Valades AG, Pozo M, Varela L, et al. Mitochondrial cristae-remodeling protein OPA1 in POMC neurons couples Ca(2+) homeostasis with adipose tissue lipolysis. Cell Metab 2021;33:1820–35 e1829. 34343501 10.1016/j.cmet.2021.07.008 PMC8432968

[71] Yi CX, Walter M, Gao Y, et al. TNFalpha drives mitochondrial stress in POMC neurons in obesity. Nat Commun 2017;8:15143. 28489068 10.1038/ncomms15143 PMC5436136

[72] Kang GM, Min SH, Lee CH, et al. Mitohormesis in hypothalamic POMC neurons mediates regular exercise-induced high-turnover metabolism. Cell Metab 2021;33:334–49 e336. 33535098 10.1016/j.cmet.2021.01.003 PMC7959183

[73] Kim SJ, Kwon MC, Ryu MJ, et al. CRIF1 is essential for the synthesis and insertion of oxidative phosphorylation polypeptides in the mammalian mitochondrial membrane. Cell Metab 2012;16:274–83. 22819524 10.1016/j.cmet.2012.06.012

[74] Timper K, Paeger L, Sánchez-Lasheras C, et al. Mild impairment of mitochondrial OXPHOS promotes fatty acid utilization in POMC neurons and improves glucose homeostasis in obesity. Cell Rep 2018;25:383–97 e310. 30304679 10.1016/j.celrep.2018.09.034 PMC6349418

[75] Jeong DE, Lee D, Hwang SY, et al. Mitochondrial chaperone HSP-60 regulates anti-bacterial immunity via p38 MAP kinase signaling. EMBO J 2017;36:1046–65. 28283579 10.15252/embj.201694781 PMC5391144

[76] Filograna R, Lee S, Tiklova K, et al. Mitochondrial dysfunction in adult midbrain dopamine neurons triggers an early immune response. PLoS Genet 2021;17:e1009822. 34570766 10.1371/journal.pgen.1009822 PMC8496783

[77] Lee H, Fenster RJ, Pineda SS, et al. Cell type-specific transcriptomics reveals that mutant huntingtin leads to mitochondrial RNA release and neuronal innate immune activation. Neuron 2020;107:891–908 e898. 32681824 10.1016/j.neuron.2020.06.021 PMC7486278

[78] Yun J, Finkel T. Mitohormesis. Cell Metab 2014;19:757–66. 24561260 10.1016/j.cmet.2014.01.011 PMC4016106

[79] Ramirez S, Gómez-Valadés AG, Schneeberger M, et al. Mitochondrial dynamics mediated by mitofusin 1 is required for POMC neuron glucose-sensing and insulin release control. Cell Metab 2017;25:1390–9 e1396. 28591639 10.1016/j.cmet.2017.05.010

[80] Zhang Q, Tian Y. Molecular insights into the transgenerational inheritance of stress memory. J Genet Genomics 2022;49:89–95. 34923165 10.1016/j.jgg.2021.11.015

[81] Zhang Q, Wang Z, Zhang W, et al. The memory of neuronal mitochondrial stress is inherited transgenerationally via elevated mitochondrial DNA levels. Nat Cell Biol 2021;23:870–80. 34341532 10.1038/s41556-021-00724-8

[82] Morais LH, Schreiber HL, Mazmanian SK. The gut microbiota-brain axis in behaviour and brain disorders. Nat Rev Microbiol 2021;19:241–55. 33093662 10.1038/s41579-020-00460-0

[83] Bove J, Prou D, Perier C, et al. Toxin-induced models of Parkinson’s disease. NeuroRx 2005;2:484–94. 16389312 10.1602/neurorx.2.3.484 PMC1144492

[84] Sherer TB, Betarbet R, Testa CM, et al. Mechanism of toxicity in rotenone models of Parkinson’s disease. J Neurosci 2003;23:10756–64. 14645467 10.1523/JNEUROSCI.23-34-10756.2003 PMC6740985

[85] Sliter DA, Martinez J, Hao L, et al. Parkin and PINK1 mitigate STING-induced inflammation. Nature 2018;561:258–62. 30135585 10.1038/s41586-018-0448-9 PMC7362342

[86] Celardo I, Martins LM, Gandhi S. Unravelling mitochondrial pathways to Parkinson’s disease. Br J Pharmacol 2014;171:1943–57. 24117181 10.1111/bph.12433 PMC3976614

[87] Palavicini JP, Chen J, Wang C, et al. Early disruption of nerve mitochondrial and myelin lipid homeostasis in obesity-induced diabetes. JCI Insight 2020;5:e137286. 33148881 10.1172/jci.insight.137286 PMC7710310

[88] Wang L, Ishihara T, Ibayashi Y, et al. Disruption of mitochondrial fission in the liver protects mice from diet-induced obesity and metabolic deterioration. Diabetologia 2015;58:2371–80. 26233250 10.1007/s00125-015-3704-7

[89] Jensen-Cody SO, Flippo KH, Claflin KE, et al. FGF21 signals to glutamatergic neurons in the ventromedial hypothalamus to suppress carbohydrate intake. Cell Metab 2020;32:273–86 e276. 32640184 10.1016/j.cmet.2020.06.008 PMC7734879

[90] Wall CE, Whyte J, Suh JM, et al. High-fat diet and FGF21 cooperatively promote aerobic thermogenesis in mtDNA mutator mice. Proc Natl Acad Sci USA 2015;112:8714–9. 26124126 10.1073/pnas.1509930112 PMC4507233

[91] Zhang Y, Xie Y, Berglund ED, et al. The starvation hormone, fibroblast growth factor-21, extends lifespan in mice. Elife 2012;1:e00065. 23066506 10.7554/eLife.00065 PMC3466591

[92] Valenzuela PL, Castillo-García A, Morales JS, et al. Exercise benefits on Alzheimer’s disease: state-of-the-science. Ageing Res Rev 2020;62:101108. 32561386 10.1016/j.arr.2020.101108

[93] Ost M, Igual Gil C, Coleman V, et al. Muscle-derived GDF15 drives diurnal anorexia and systemic metabolic remodeling during mitochondrial stress. EMBO Rep 2020;21:e48804. 32026535 10.15252/embr.201948804 PMC7054681

[94] Tsai VW, Manandhar R, Jørgensen SB, et al. The anorectic actions of the TGFbeta cytokine MIC-1/GDF15 require an intact brainstem area postrema and nucleus of the solitary tract. PLoS One 2014;9:e100370. 24971956 10.1371/journal.pone.0100370 PMC4074070

[95] Tsai VW, Zhang HP, Manandhar R, et al. GDF15 mediates adiposity resistance through actions on GFRAL neurons in the hindbrain AP/NTS. Int J Obes (Lond) 2019;43:2370–80. 31152154 10.1038/s41366-019-0365-5

[96] Burtscher J, Millet GP, Place N, et al. The muscle-brain axis and neurodegenerative diseases: the key role of mitochondria in exercise-induced neuroprotection. Int J Mol Sci 2021;22:6479. 34204228 10.3390/ijms22126479 PMC8235687

[97] Calculli G, Lee HJ, Shen K, et al. Systemic regulation of mitochondria by germline proteostasis prevents protein aggregation in the soma of C. elegans. Sci Adv 2021;7:eabg3012. 34172445 10.1126/sciadv.abg3012 PMC8232903

[98] Shen K, Pender CL, Bar-Ziv R, et al. Mitochondria as cellular and organismal signaling hubs. Annu Rev Cell Dev Biol 2022;38:179–218. 35804477 10.1146/annurev-cellbio-120420-015303

[99] Dutta N, Garcia G, Higuchi-Sanabria R. Hijacking cellular stress responses to promote lifespan. Front Aging 2022;23:860404. 10.3389/fragi.2022.860404 PMC9261414 35821861

[100] Zhang Q, Wu X, Chen P, et al. The mitochondrial unfolded protein response is mediated cell-non-autonomously by retromer-dependent Wnt signaling. Cell 2018;174:870–83 e817. 30057120 10.1016/j.cell.2018.06.029 PMC6086732

[101] Li X, Li J, Zhu D, et al. Protein disulfide isomerase PDI-6 regulates Wnt secretion to coordinate inter-tissue UPR(mt) activation and lifespan extension in C. elegans. Cell Rep 2022;39:110931. 35675782 10.1016/j.celrep.2022.110931

[102] Fisher FM, Maratos-Flier E. Understanding the physiology of FGF21. Annu Rev Physiol 2016;78:223–41. 26654352 10.1146/annurev-physiol-021115-105339

[103] Kang SG, Choi MJ, Jung SB, et al. Differential roles of GDF15 and FGF21 in systemic metabolic adaptation to the mitochondrial integrated stress response. iScience 2021;24:102181. 33718833 10.1016/j.isci.2021.102181 PMC7920832

[104] Cobb LJ, Lee C, Xiao J, et al. Naturally occurring mitochondrial-derived peptides are age-dependent regulators of apoptosis, insulin sensitivity, and inflammatory markers. Aging 2016;8:796–809. 27070352 10.18632/aging.100943 PMC4925829

[105] Kim KH, Son JM, Benayoun BS, et al. The mitochondrial-encoded peptide MOTS-c translocates to the nucleus to regulate nuclear gene expression in response to metabolic stress. Cell Metab 2018;28:516–24 e517. 29983246 10.1016/j.cmet.2018.06.008 PMC6185997

[106] Kovaleva IE, Garaeva AA, Chumakov PM, et al. Evstafieva, Intermedin/adrenomedullin 2 is a stress-inducible gene controlled by activating transcription factor 4. Gene 2016;590:177–85. 27328454 10.1016/j.gene.2016.06.037

[107] Oike Y, Akao M, Yasunaga K, et al. Angiopoietin-related growth factor antagonizes obesity and insulin resistance. Nat Med 2005;11:400–8. 15778720 10.1038/nm1214

[108] Nashine S, Cohen P, Nesburn AB, et al. Characterizing the protective effects of SHLP2, a mitochondrial-derived peptide, in macular degeneration. Sci Rep 2018;8:15175. 30310092 10.1038/s41598-018-33290-5 PMC6182005

[109] Chandel NS. Evolution of mitochondria as signaling organelles. Cell Metab 2015;22:204–6. 26073494 10.1016/j.cmet.2015.05.013

